# ﻿Current and future potential distribution of two bamboo pests in China: *Anakaburmensis* and *Cicadellaviridis* (Hemiptera, Cicadellidae)

**DOI:** 10.3897/zookeys.1203.118978

**Published:** 2024-05-30

**Authors:** Zhengxue Zhao, Lin Yang, Xiangsheng Chen

**Affiliations:** 1 Institute of Entomology, Guizhou University, Guiyang, China Guizhou University Guiyang China; 2 Provincial Special Key Laboratory for Development and Utilization of Insect Resources of Guizhou, Guizhou University, Guiyang, China Anshun University Anshun China; 3 Guizhou Key Laboratory for Agricultural Pest Management of Mountainous Region, Guizhou University, Guiyang, China Guizhou University Guiyang China; 4 College of Agriculture, Anshun University, Anshun, China Anshun University Anshun China

**Keywords:** Climate change, distribution areas, Maxent model

## Abstract

China’s bamboo output is closely associated with its national economy; however, it is currently rapidly declining due to damage from the pests *Anakaburmensis* and *Cicadellaviridis*. Identifying regions that are environmentally suitable for these pests is a critical step in their effective control. Therefore, in this study, we used a Maxent model to predict their current and future potential areas of distribution (2021–2040, 2041–2060, and 2061–2080) and explored changes over time using distribution data and related environmental variables. The model results demonstrates that the current potential areas of distribution of *A.burmensis* are predominantly concentrated in several provinces of southern and central China, such as Guizhou, Guangxi, and Hubei, whereas the current potential areas of distribution of *C.viridis* are primarily in many provinces across southern, central, and northeastern China. In the future, the potential distribution of *A.burmensis* will increase and move minimally, whereas the potential distribution of *C.viridis* will decrease and move considerably. The results of the present study provide vital information for predicting the spread and outbreaks of *C.viridis* and *A.burmensis* and provide a reference framework for developing management strategies to control these two pests, thereby minimizing economic loss in the bamboo industry.

## ﻿Introduction

Bamboo comprises all members of the subfamily Bambusoideae and is the only lineage in the Poaceae family that has adapted and diversified to forest habitats (Judziewicz et al. 1999; Grass Phylogeny Working Group 2001; Bamboo Phylogeny Group 2012). Bamboo plants are mainly distributed in the tropical and subtropical regions of Asia, Africa, and Latin America (Li et al. 2003). The emergence of bamboo has provided several benefits to humans (Sharma et al. 2014). For example, bamboo is a suitable substitute for wood due to its advantages of a short rotation period, strong regeneration ability, good properties for wide use, and excellent performance; these properties are similar to or even superior to those of wood (Yang et al. 2004). Therefore, a growing number of countries, and especially China, use bamboo as a common building material to save wood (Van der Lugt et al. 2006; Sharma et al. 2014). Bamboo also plays a crucial role in combating degradation of mountain environments, ecosystems, and natural resources (Yang et al. 2004). Furthermore, young, tender bamboo shoots are used as a seasonal vegetable in both rural and urban areas of China (Borah et al. 2008). However, unfortunately, bamboo is vulnerable to damage from herbivorous insect pests throughout its lifetime; thus, pests are the main cause of the massive loss of bamboo in natural and plantation forests (Liese and Köhl 2015).

China has abundant bamboo resources, with 753 species accounting for approximately 50% of the world’s bamboo (Shi et al. 2020). Bamboo is widely distributed in China and is found in 27 of the 34 provincial administrative regions (Shu et al. 2015). In China, the output value of bamboo reaches 45 billion yuan per year, which has contributed significantly to China’s economic development (Shu et al. 2015). However, in recent years, due to the emergence of numerous bamboo pests, bamboo production has declined sharply (Yang et al. 2011; Shu et al. 2015), causing serious economic losses. Therefore, strengthening the management and control of bamboo pests is conducive to the steady growth of China’s economy.

*Anakaburmensis* Dworakowska, 1993 and *Cicadellaviridis* (Linnaeus, 1758) are two important bamboo pests (Xu and Wang 2004; Yang et al. 2007; Dong et al. 2017). Both pests are tiny insects, with body sizes of 3.45–4.05 and 6.2–10.8 mm, respectively (Chen et al. 2012). Owing to this characteristic, they can only be observed when they congregate. Both pests have varying feeding behavior. *Anakaburmensis* feeds on the content of the cells, resulting in the formation of “white spots” or “hopper burn” on the leaf surface. *Cicadellaviridis* is a xylem feeder and causes no considerable damage to the leaf surface. They prefer feeding on the stem or larger veins of the plant where they can easily reach the xylem tubes.

*Anakaburmensis* is distributed across southern China, including Guizhou and Yunnan (Dong et al. 2017), whereas *C.viridis* is more widely distributed in northern China (Liu et al. 2004; Lin et al. 2016; Zhao et al. 2022). Determining the distributional range of a pest is essential for its control; however, the distribution of these two pests has not yet been fully investigated. Traditional manual surveys cannot completely determine the distribution of pests in large geographical areas, such as China, due to limited resources and failure to predict the distributional range of species due to climate change. This frequently results in the use of pest control measures during outbreaks, causing large economic losses. Therefore, preventive methods are urgently required to solve this problem.

Species distribution models use occurrence records and environmental data to produce a model of the species’ requirements and a map of its potential distribution (Anderson et al. 2002). Currently, species distribution models have been widely recognized as a useful tool for predicting pest distribution areas (Geng et al. 2011; Kumar et al. 2016; Huang et al. 2019; Xu et al. 2020). Among species distribution models, the Maxent model is the most commonly used, largely because it has significant advantages of requiring only a small sample size and having good performance compared with other models. Phillips et al. (2006) compared the performance of Maxent and GARP (Genetic Algorithm for Ruleset Prediction) models based on 10 random subsets of the occurrence records for two Neotropical mammal species and found that the Maxent model was almost always higher, suggesting that it better predicted species distribution than the GARP model.

In this study, we used a Maxent model to predict the current and future potential distribution areas of *A.burmensis* and *C.viridis* in China using occurrence records and environmental data. We aim to answer two questions: 1) where are the potential distribution areas, both currently and in the future, and 2) how has the potential distribution area changed over time?

## ﻿Materials and methods

### ﻿Occurrence records

**Figure 1.**
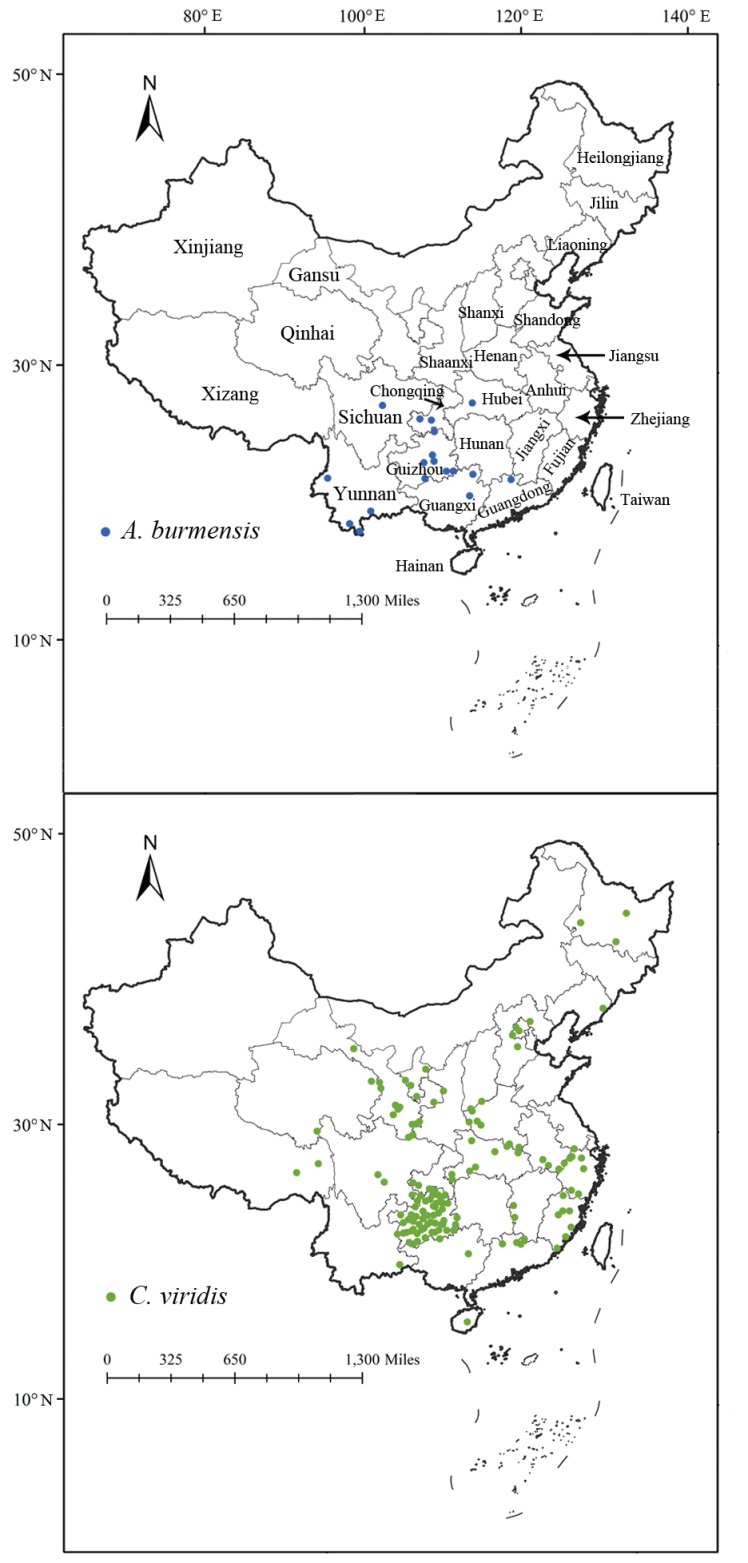
Occurrence of *Anakaburmensis* and *Cicadellaviridis* in China.

We collected the occurrence records of *Anakaburmensis* and *Cicadellaviridis* in China from the literature and from the Global Biodiversity Information Facility (GBIF, https://www.gbif.org/). Occurrence records that lacked latitude and longitude data were georeferenced using Google Earth. Sampling bias in geographic space frequently arises due to unequal sampling efforts, which can lead to incorrect predictions in species distribution models (Kramer-Schadt et al. 2013; Boria et al. 2014; Fourcade et al. 2014). Therefore, we conducted spatial thinning for the occurrence records with a 10-km distance using the R software package “spThin” to reduce the effect of sampling bias (Aiello-Lammens et al. 2015). After spatial thinning, 18 and 135 occurrence records of *A.burmensis* and *C.viridis* were obtained, respectively (Fig. 1, Suppl. material 1: table S1).

### ﻿Environmental variables

We used 19 bioclimatic variables (1970–2000) and one elevation datum (altitude) from the current period as the current environment variables. To minimize multicollinearity among environmental variables, we calculated the variance inflation factor for the corresponding environmental variable values of the occurrence records of *A.burmensis and C.viridis*. Then, we eliminated the environment variable with the largest variance inflation factor each time until the variance inflation factor values of selected environment variables were less than 5. Finally, four environmental variables were retained for *A.burmensis*: mean diurnal range (bio2), mean temperature of wettest quarter (bio8), precipitation of warmest quarter (bio18), and precipitation of coldest quarter (bio19), whereas five environmental variables were retained for *C.viridis*: isothermality (bio3), mean temperature of wettest quarter (bio8), precipitation of driest month (bio14), precipitation seasonality (bio15), and precipitation of warmest quarter (bio18).

We selected future bioclimatic variables for the periods 2021–2040, 2041–2060, and 2061–2080 from the Coupled Model Intercomparison Project Phase 6 (CMIP 6). Four main emission scenarios driven by different socioeconomic assumptions are provided for CMIP6: SSP126, SSP245, SSP370, and SSP585. However, to avoid extreme simulation, the SSP245 scenario, a moderate emission scenario, was used (Hwang et al. 2022). In addition, due to the uncertainty of future climate models, future bioclimatic variables of the SSP245 scenario were obtained by calculating the mean from CanESM5, IPSL-CM6A-LR, and MIROC6 models (Zhao et al. 2024).

The bioclimatic variables and elevation data were obtained from the WorldClim database (http://www.worldclim.org), with a 2.5-arc min spatial resolution (~5 km resolution at the equator).

### ﻿Maxent model

The model was developed in Maxent software v. 3.4.4. Regular multipliers (RM) and feature classes (FC) are closely related to the accuracy of the Maxent model (Phillips and Dudík 2008). Therefore, we used the ENMeval package in R software to choose the best combination of FC and RM values based on the lowest Akaike’s Information Corrected Criterion (AICc) score (Muscarella et al. 2014). The RM values ranged from 1 to 4 in increments of 1, while eight FC were used: (i) Linear (L); (ii) Linear and Quadratic (LQ); (iii) Linear, Quadratic, and Hinge (LQH); (iv) Hinge, Product, and Threshold (HPT); (v) Quadratic, Hinge, and Product (QHP); (vi) Linear, Quadratic, Hinge, and Product (LQHP); (vii) Quadratic, Hinge, Product, and Threshold (QHPT); (viii) Linear, Quadratic, Hinge, Product, and Threshold (LQHPT). After the above process, an RM of 3 and the FCLQH were selected for *A.burmensis* and an RM of 1 and the FCLQ were selected for *C.viridis*. In addition, 10,000 background points, five repetitions with cross validation, and logistic output were set to run the model. The performance of the Maxent model was evaluated based on the area under the receiver operating characteristic curve (AUC) and true skill statistic (TSS). The AUC and TSS values greater than 0.75 and 0.6 respectively were considered useful (Elith 2002; Ben Rais Lasram et al. 2010). The importance of environmental variables was measured using the jackknife method. The 10^th^ percentile training presence logistic threshold was used to define the potential distribution areas (Bosso et al. 2016; Fan et al. 2020; Wei et al. 2020; Wei et al. 2021).

### ﻿Shift in potential distribution areas

To quantify the distributional shifts between the current and future potential distribution areas, centroid analysis was performed using the SDMtoolbox 2.0 tool. This analysis converts the species distribution to a central point (centroid) and creates a vector to describe the direction and magnitude of the change through time (Brown 2014). We obtained the distribution shift by tracking the change of the centroid.

## ﻿Results

### ﻿Model validation and important variables

**Figure 2.**
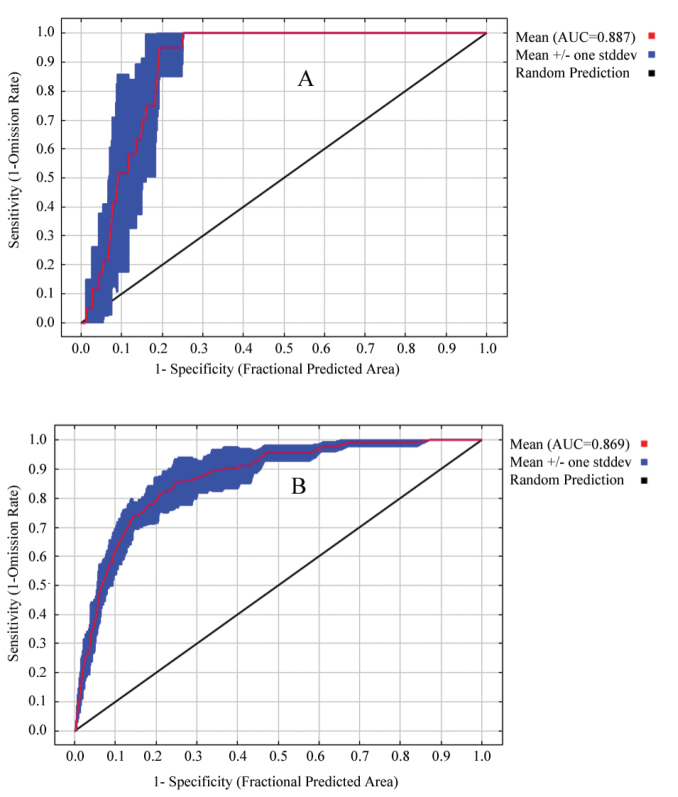
Receiver operating characteristic curve for *Anakaburmensis* (**A**) and *Cicadellaviridis* (**B**).

**Figure 3.**
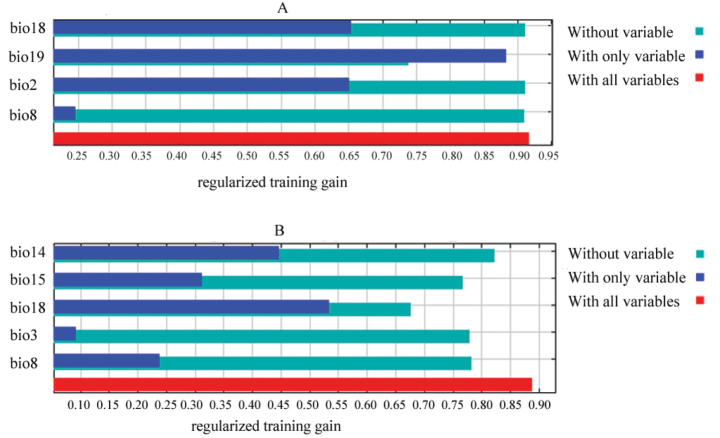
The importance of environmental variables for *Anakaburmensis* (**A**) and *Cicadellaviridis* (**B**) based on the jackknife test.

The results showed that the mean test AUC for *Anakaburmensis* and *Cicadellaviridis* were 0.887 and 0.869, respectively (Fig. 2). Moreover, mean TSS values for *A.burmensis* and *C.viridis* were 0.691 and 0.614, respectively. The jackknife test revealed that the most important variable that affected the distribution of *A.burmensis* was precipitation in the coldest quarter (bio19), followed by precipitation in the warmest quarter (bio18) (Fig. 3). The mean temperature of the wettest quarter (bio8) was found to be slightly less important than the precipitation in the warmest quarter (bio18). The mean diurnal range (bio2) had the lowest contribution to the distribution of *A.burmensis*. The most important variable that affected the distribution of *C.viridis* was found to be precipitation in the warmest quarter (bio18), followed by precipitation in the driest month (bio14) (Fig. 3). The mean temperature of the wettest quarter (bio8) and precipitation seasonality (bio15) were found to have moderate impacts. The variable with the lowest impact was isothermality (bio3) (Fig. 3).

### ﻿Potential distribution areas and changes

**Figure 4.**
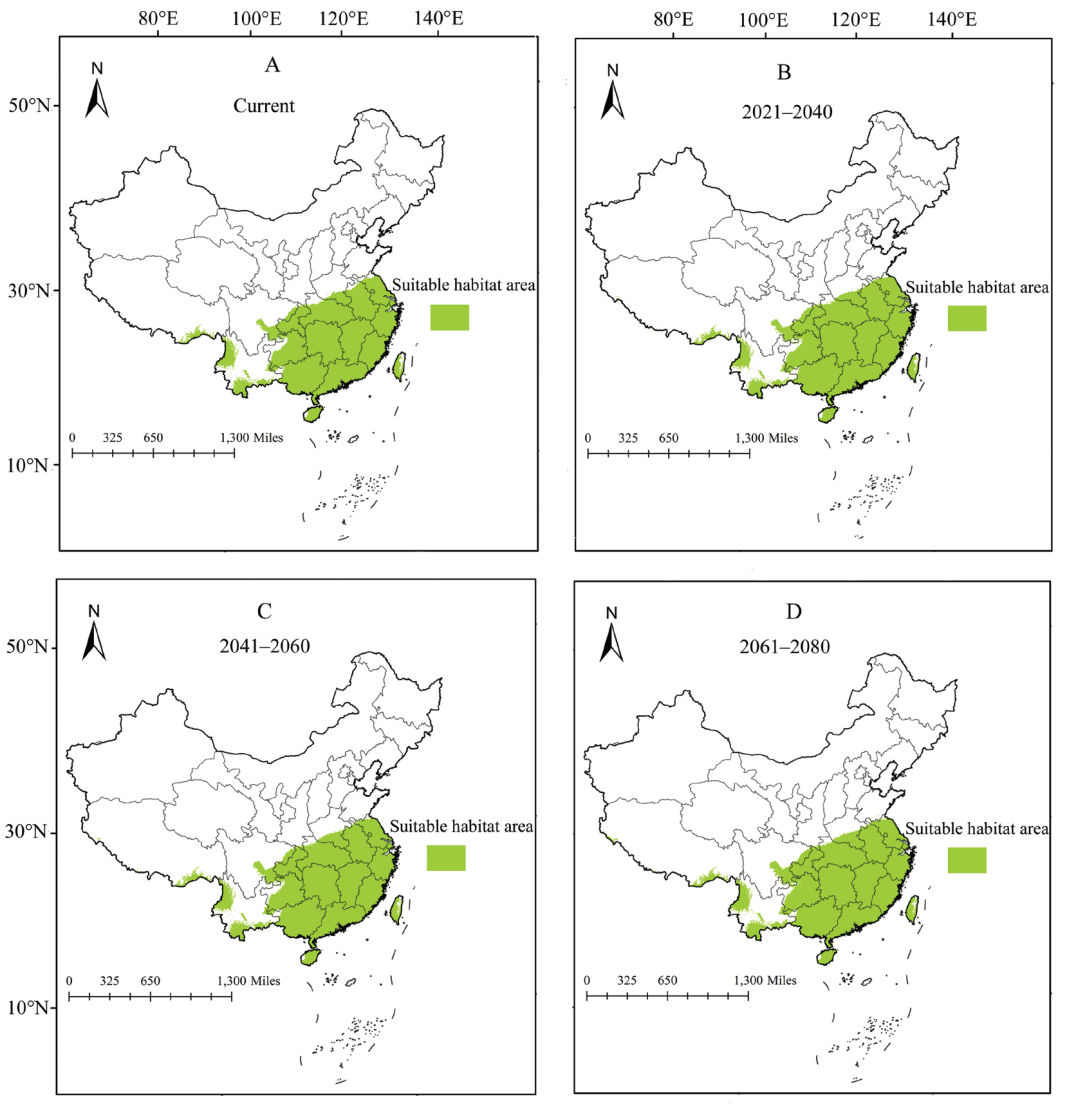
Potential distribution areas of *Anakaburmensis* in China during different periods.

**Figure 5.**
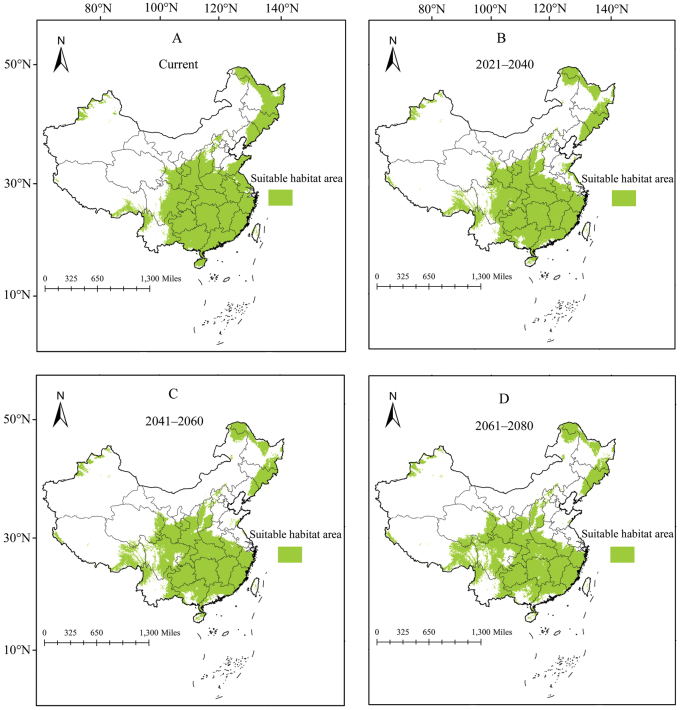
Potential distribution areas of *Cicadellaviridis* in China during different periods.

**Figure 6.**
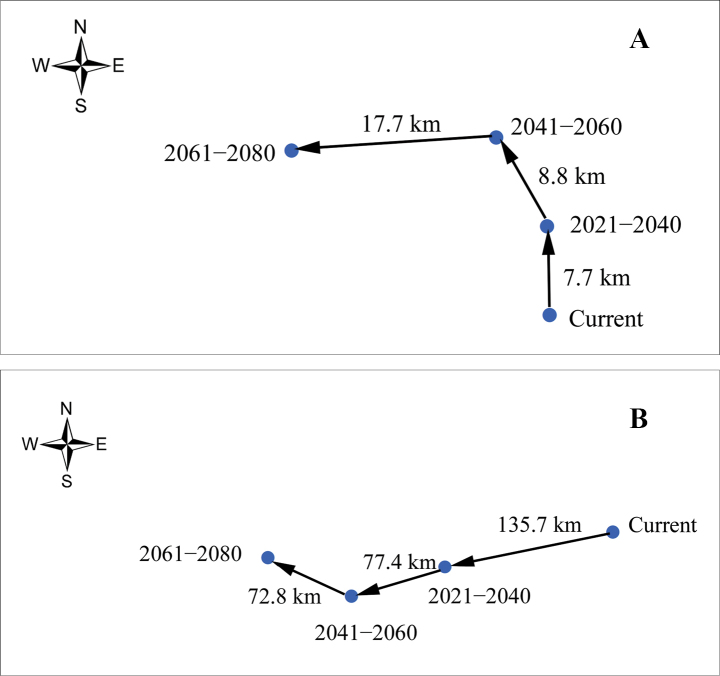
Shift in potential distribution areas of *Anakaburmensis* (**A**) and *Cicadellaviridis* (**B**) in China.

The current and future potential distribution area for two pests in China was obtained by Maxent model (Figs 4, 5). The current potential distribution area for *A.burmensis* was predicted to be 2.29 × 10^6^ km^2^ (Table 1) and was mainly concentrated in provincial administrative divisions of southern and central China (Fig. 4), including Guizhou, Chongqing, Guangxi, Hunan, Hubei, Guangdong, Jiangxi, Fujian, Anhui, Zhejiang, and Jiangsu. Potential distribution areas have also emerged in Xizang, Sichuan, Yunnan, Hainan, and Taiwan (Fig. 4). Three future periods showed increases in the size of predicted distribution area for *A.burmensis*, with increases of 2.62%, 3.49%, and 5.67% in 2021–2040, 2041–2060, and 2061–2080, respectively (Table 1). Centroid analysis revealed that centroids representing potential distribution areas moved 7.7 km north in 2021–2040, 8.8 km northwest in 2041–2060, and 17.7 km west in 2061–2080 (Fig. 6). This result revealed a slight shift in potential distribution areas.

The current potential distribution areas for *C.viridis* in China are projected to be 4.42 × 10^6^ km^2^ (Table 1), an area which is larger than that projected for *A.burmensis*. Numerous provincial administrative divisions in central, southern, and northeastern China have become major potential distribution areas for *C.viridis*, such as Guizhou, Hunan, Hubei, and Heilongjiang (Fig. 5). The future potential distribution areas were found to be decreased compared with the current areas by 5.88% in 2021–2040, 7.69% in 2041–2060, and 8.37% in 2061–2080 (Table 1). Overall, potential distribution areas for *C.viridis* are predicted to move toward the southwest in 2021–2040 (135.7 km) and 2041–2060 (77.4 km) and toward the northwest in 2061–2080 (72.8 km) due to climate change (Fig. 6).

## ﻿Discussion

In the present study, the Maxent model was used to predict the current and future potential distribution areas of two pests that seriously harm bamboo in China. In addition, the spatial variation of potential distribution areas over time was investigated. The mean AUC and TSS value of five runs of the Maxent model for the two pests was high, suggesting that the constructed models have good performance and usefulness.

This study revealed that precipitation is the most important environmental factor driving the distribution of *Anakaburmensis* and *Cicadellaviridis*. This is consistent with the findings of previous studies on other pests, such as *Moritziellacastaneivora* Miyazaki, 1968 (Wang et al. 2010), *Riptortuspedestris* (Fabricius, 1775) (Zhang et al. 2022), and *Spodopterafrugiperda* (Smith, 1797) (Ramasamy et al. 2022). Therefore, the results of the current and previous studies suggest that precipitation is a key factor for pest distribution. Furthermore, the current potential distribution areas of *A.burmensis* and *C.viridis*, as predicted by the Maxent model, were not only present in these provinces with occurrence records but also in several other provinces. This result largely reflects serious insufficiency in the current field investigation for the two pests and suggests that the Maxent model can be used as a pest monitoring tool.

The main distribution range of the current and future potential distribution areas of *A.burmensis* and *C.viridis* is the main distribution area of bamboo in China (Xu et al. 2019), which implies that bamboo in China is potentially threatened by these two pests. The potential distribution areas and the spatial change of *A.burmensis* and *C.viridis* identified in this study are extremely important in providing guidance for the management of these pests. For instance, management efforts for *A.burmensis* should continue to be focused on the southern and central China due to insignificant changes in the future. Moreover, the potential distribution areas for *C.viridis* appear to have a slight reduction in the future compared with current, but anticipated expansion of potential distribution areas in Sichuan, Qinhai, and Xizang is predicted; therefore, it is vital to develop effective preemptive strategies (e.g. strict quarantine measures) to prevent the introduction of this pest into these regions.

Zhang et al. (2023) used a Maxent model to predict current and future potential distribution areas of *C.viridis* globally. In their study, many areas in China were found to be environmentally suitable for this pest, but there was a huge difference from our prediction; the distribution of potential distribution areas found in our study revealed a greater range, with northeast Liaoning and northeast Heilongjiang becoming potential distribution. Wei et al. (2023) also obtained potential distribution areas of *C.viridis* in China using the Maxent model and found that most areas have become environmentally suitable. Although the abovementioned two studies have identified potential distribution areas in China, there are common or unique limitations in the modelling process, such as the use of untuned key parameters (i.e., FC and RM) for two studies and outdated future climate data in the study of Wei et al. (2023). Consequently, prediction results of the two studies are highly likely to be biased. Objectively, our results are more accurate due to the use of a more correct modeling process.

Although this study forecasted potential distribution areas for the two pests, the results must be interpreted with caution and some limitations should be acknowledged. All data for the modelling process were derived from GBIF and the literature. It is important to supplement these data with field investigation in future studies and perform the test of prediction. For example, by going to the field where the distribution of the species is predicted, we can confirm that the species is found in the field. Moreover, species distribution is determined by three factors: (1) the capacity to reach a suitable location; (2) the capacity to develop in a certain environmental condition; and (3) the ability to compete with other species occupying the same region (Begon et al. 2005). However, in our study, we only considered the effect of climate on species distribution. Furthermore, the physiological needs of species are plastic and may change over the course of evolution (Thomson et al. 2010), a fact that should be considered in future research.

## ﻿Conclusions

In summary, the current and future potential distribution areas of *Anakaburmensis* and *Cicadellaviridis* in China were obtained using the Maxent model. The results of this study demonstrated that precipitation is the most important environmental factor in shaping the distribution of these two pests. In addition, the findings of this study will assist policymakers and governments in developing appropriate measures for managing and controlling *A.burmensis* and *C.viridis*, thereby decreasing the damage to bamboo and the associated significant economic loss.

## ﻿Additional information

### Author ORCIDs

Zhengxue Zhao https://orcid.org/0000-0003-1764-8690

### Data availability

All of the data that support the findings of this study are available in the main text or Supplementary Information.

## Supplementary materials

Supplementary material 1 Supplemenatry data Data type xlsx https://binary.pensoft.net/file/1059320This dataset is made available under the Open Database License (http://opendatacommons.org/licenses/odbl/1.0/). The Open Database License (ODbL) is a license agreement intended to allow users to freely share, modify, and use this Dataset while maintaining this same freedom for others, provided that the original source and author(s) are credited.Zhengxue Zhao, Lin Yang, Xiangsheng Chen

## References

[B1] Aiello-Lammens ME Boria RA Radosavljevic A Vilela B Anderson RB (2015) spThin: An R package for spatial thinning of species occurrence records for use in ecological niche models. Ecography 38(5): 541–545. 10.1111/ecog.01132

[B2] Anderson RP Góme-Laverde M Peterson AT (2002) Geographical distributions of spiny pocket mice in South America: Insights from predictive models. Global Ecology and Biogeography 11(2): 131–141. 10.1046/j.1466-822X.2002.00275.x

[B3] Bamboo Phylogeny Group (2012) An updated tribal and subtribal classification of the bamboos (Poaceae: Bambusoideae). In: Gielis J, Potters G (Eds) Proceedings of the 9^th^ World Bamboo Congress, Antwerp, Belgium.

[B4] Begon M Townsend CR Harper JL (2005) Ecology: from individuals to ecosystems, 4^th^ edition. Blackwell Publishing, Malden.

[B5] Ben Rais Lasram F Guilhaumon F Albouy C Somot S Thuiller W Mouillot D (2010) The Mediterranean Sea as a ‘cul‐de‐sac’for endemic fishes facing climate change. Global Change Biology 16(12): 3233–3245. 10.1111/j.1365-2486.2010.02224.x

[B6] Borah ED Pathak KC Deka B Neog D Borah K (2008) Utilization aspects of Bamboo and its market value. Indian Forester 134: 423–427.

[B7] Boria RA Olson LE Goodman SM Anderson RP (2014) Spatial filtering to reduce sampling bias can improve the performance of ecological niche models. Ecological Modelling 275: 73–77. 10.1016/j.ecolmodel.2013.12.012

[B8] Bosso L Di Febbraro M Cristinzio G Zoina A Russo D (2016) Shedding light on the effects of climate change on the potential distribution of *Xylellafastidiosa* in the Mediterranean basin. Biological Invasions 18(6): 1759–1768. 10.1007/s10530-016-1118-1

[B9] Brown JL (2014) SDMtoolbox, a python-based GIS toolkit for landscape genetic, biogeographic and species distribution model analyses. Methods in Ecology and Evolution 5(7): 694–700. 10.1111/2041-210X.12200 PMC5721907 29230356

[B10] Chen XS Yang L Li ZZ (2012) Bamboo-feeding leafhoppers in China. China Forestry Publishing House, Beijing.

[B11] Dong MS Yang L Chen XS (2017) Investigation on the distribution and damage of the Bamboo leafhopper pest, *Anakaburmensis* Dworakowska (Hemiptera: Cicadellidae) in China. Journal of Mountain Agriculture and Biology 36: 031–034.

[B12] Elith J (2002) Quantitative methods for modeling species habitat: comparative performance and an application to Australian plants. In: Ferson S, Burgman M (Eds) Quantitative Methods for Conservation Biology. Springer.

[B13] Fan S Chen C Zhao Q Wei J Zhang H (2020) Identifying potentially climatic suitability areas for *Armacustos* (Hemiptera: Pentatomidae) in China under climate change. Insects 11(10): 674. 10.3390/insects11100674 33020387 PMC7600814

[B14] Fourcade Y Engler JO Rodder D Secondi J (2014) Mapping species distributions with MAXENT using a geographically biased sample of presence data: A performance assessment of methods for correcting sampling bias. PLoS One 9(5): e97122. 10.1371/journal.pone.0097122 PMC4018261 24818607

[B15] Geng J Li ZH Rajotte EG Wan FH Lu XY Wang ZL (2011) Potential geographical distribution of *Rhagoletispomonella* (Diptera: Tephritidae) in China. Insect Science 18(5): 575–582. 10.1111/j.1744-7917.2010.01402.x

[B16] Grass Phylogeny Working Group (2001) Phylogeny and subfamilial classification of the grasses (Poaceae). Annals of the Missouri Botanical Garden 88(3): 373–457. 10.2307/3298585

[B17] Huang M Ge X Shi H Tong Y Shi J (2019) Prediction of current and future potential distributions of the Eucalyptus pest *Leptocybeinvasa* (Hymenoptera: Eulophidae) in China using the CLIMEX model. Pest Management Science 75(11): 2958–2968. 10.1002/ps.5408 30868710

[B18] Hwang JH Kim SH Yoon S Jung S Kim DH Lee WH (2022) Evaluation of spatial distribution of three major *Leptocorisa* (Hemiptera: Alydidae) pests using Maxent model. Insects 13(8): 750. 10.3390/insects13080750 36005375 PMC9409444

[B19] Judziewicz EJ Clark LG London X Stern MJ (1999) American Bamboos. Smithsonian Institution Press, Washington DC.

[B20] Kramer-Schadt S Niedballa J Pilgrim JD Schröder B Lindenborn J Reinfelder V Stillfried M Heckmann I Scharf AK Augeri DM Cheyne SM Hearn AJ Ross J Macdonald DW Mathai J Eaton J Marshall AJ Semiadi G Rustam R Bernard H Alfred R Samejima H Duckworth JW Breitenmoser-Wuersten C Belant JL Hofer H Wilting A (2013) The importance of correcting for sampling bias in MaxEnt species distribution models. Diversity & Distributions 19(11): 1366–1379. 10.1111/ddi.12096

[B21] Kumar S Yee WL Neven LG (2016) Mapping global potential risk of establishment of *Rhagoletispomonella* (Diptera: Tephritidae) using Maxent and CLIMEX niche models. Journal of Economic Entomology 109(5): 2043–2053. 10.1093/jee/tow166 27452001

[B22] Li R Zhang J Zhang ZE (2003) Values of bamboo biodiversity and its protection in China. Journal of Bamboo Research 22: 7–17.

[B23] Liese W Köhl M (2015) Bamboo: the Plant and its Uses. Springer, Berlin. 10.1007/978-3-319-14133-6

[B24] Lin SY Cao WQ Wang YQ Hu HY (2016) Primary investigation and study of chalcidoid wasps resources in Xinjiang Manas National Wetland Park where *Cicadellaviridis* breaks out. Xinjiang Nongye Kexue 53: 1850–1857.

[B25] Liu YJ Mu YJ Wang CY (2004) Spatial distribution of mark of ova of *Cicadellaviridis* in fruit trees and its application. Inner Mongolia Forestry Science and Technology 5: 22–24.

[B26] Muscarella R Galante PJ Soley-Guardia M Boria RA Kass JM Uriarte M Anderson RP McPherson J (2014) ENMeval: An R package for conducting spatially independent evaluations and estimating optimal model complexity for Maxent ecological niche models. Methods in Ecology and Evolution 5(11): 1198–1205. 10.1111/2041-210X.12261

[B27] Phillips SJ Dudík M (2008) Modeling of species distributions with Maxent new extensions. Ecography 31(2): 161–175. 10.1111/j.0906-7590.2008.5203.x

[B28] Phillips SJ Anderson RP Schapire RE (2006) Maximum entropy modeling of species geographic distributions. Ecological Modelling 190(3–4): 231–259. 10.1016/j.ecolmodel.2005.03.026

[B29] Ramasamy M Das B Ramesh R (2022) Predicting climate change impacts on potential worldwide distribution of fall armyworm based on CMIP6 projections. Journal of Pest Science 95(2): 841–854. 10.1007/s10340-021-01411-1

[B30] Sharma P Dhanwantri K Mehta SDKM (2014) Bamboo as a building material. International Journal of Civil Engineering Research 5: 249–254.

[B31] Shi JY Zhou DQ Ma LS Yao J Zhang DM (2020) Diversity and important value of bamboos in China. World Bamboo Rattan 18: 55–72.

[B32] Shu JP Ye BH Wu X Zhang Y Wang H Xu T (2015) Research advances in bamboo pests and their control measures. World Forestry Research 8: 20–57.

[B33] Thomson LJ Macfadyen S Hoffmann AA (2010) Predicting the effects of climate change on natural enemies of agricultural pests. Biological Control 52(3): 296–306. 10.1016/j.biocontrol.2009.01.022

[B34] Van der Lugt P Van den Dobbelsteen AAJF Janssen JJA (2006) An environmental, economic and practical assessment of bamboo as a building material for supporting structures. Construction & Building Materials 20(9): 648–656. 10.1016/j.conbuildmat.2005.02.023

[B35] Wang XY Huang XL Jiang LY Qiao GX (2010) Predicting potential distribution of chestnut phylloxerid (Hemiptera: Phylloxeridae) based on GARP and Maxent ecological niche models. Journal of Applied Entomology 134(1): 45–54. 10.1111/j.1439-0418.2009.01447.x

[B36] Wei J Peng L He Z Lu Y Wang F (2020) Potential distribution of two invasive pineapple pests under climate change. Pest Management Science 76(5): 1652–1663. 10.1002/ps.5684 31724310

[B37] Wei J Gao G Wei JF (2021) Potential global distribution of *Daktulosphairavitifoliae* under climate change based on MaxEnt. Insects 12(4): 347. 10.3390/insects12040347 33924706 PMC8069807

[B38] Wei XJ Xu DP Zhuo ZH (2023) Predicting the impact of climate change on the geographical distribution of leafhopper, *Cicadellaviridis* in China through the MaxEnt model. Insects 14(7): 586. 10.3390/insects14070586 37504592 PMC10380802

[B39] Xu TS Wang HJ (2004) Main Pests of Bamboo in China. Chinese Forest Publish House, Beijing.

[B40] Xu Y Shen ZH Ying LX Zang RG Jiang YX (2019) Effects of current climate, paleo-climate, and habitat heterogeneity in determining biogeographical patterns of evergreen broad-leaved woody plants in China. Journal of Geographical Sciences 29(7): 1142–1158. 10.1007/s11442-019-1650-x

[B41] Xu D Li X Jin Y Zhuo Z Yang H Hu J Wang RL (2020) Influence of climatic factors on the potential distribution of pest *Heortiavitessoides* Moore in China. Global Ecology and Conservation 23: e01107. 10.1016/j.gecco.2020.e01107

[B42] Yang YM Wang KL Pei SJ Hao JM (2004) Bamboo diversity and traditional uses in Yunnan, China. Mountain Research and Development 24(2): 157–165. 10.1659/0276-4741(2004)024[0157:BDATUI]2.0.CO;2

[B43] Yang L Li ZZ Jin X (2007) *Anakaburmensis* Dworakowska: a new record leafhopper attacking bamboo (Hemiptera: Cicadellidae: Typhlocybinae) from China. Journal of Mountain Agriculture and Biology 26: 444–447.

[B44] Yang L Li ZZ Chen XS (2011) Research progress on bamboo-feeding leafhoppers. Hubei Agricultural Sciences 50: 2386–2388.

[B45] Zhang HF Wang Y Wang ZB Ding WL Xu KD Li LL Wang YY Li JB Yang M Liu X Huang X (2022) Modelling the current and future potential distribution of the bean bug *Riptortuspedestris* with increasingly serious damage to soybean. Pest Management Science 78(10): 4340–4352. 10.1002/ps.7053 35754391

[B46] Zhang YB Zhao ZX Wang YJ Liu TL (2023) Suitable habitats for *Cicadellaviridis* and *Evacanthusinterruptus* (Hemiptera: Cicadellidae) with global climate change. Journal of Entomological Science 58(2): 215–229. 10.18474/JES22-36

[B47] Zhao Q Lin SY Zhulidezi AS Wen W Hu HY (2022) Occurrence of *Cicadellaviridis* and the biology of its egg parasitoids in Xinjiang. Zhongguo Shengwu Fangzhi Xuebao 38: 29–41.

[B48] Zhao ZX Yang L Long JK Chang ZM Chen XS (2024) Predicting suitable areas for *Metcalfapruinosa* (Hemiptera: Flatidae) under climate change and implications for management. Journal of Insect Science 24(3): ieae053. 10.1093/jisesa/ieae053 PMC11078062 38717262

